# Effects of the AI-HEALS intervention based on the Fogg behavior model on self-management in postoperative breast cancer patients: a randomized controlled trial study protocol

**DOI:** 10.3389/fpubh.2026.1909032

**Published:** 2026-10-09

**Authors:** Lidan Heng, Jing Wang, Siyu Chen, Zixu Zhang, Wei Zhang, Lihua Nie, Weina Lv, Dandan Yang, Sen Li, Huitao Wang

**Affiliations:** 1 School of Nursing, Henan University of Science and Technology, Luoyang, Henan, China; 2 School of Public Health, Peking University, Beijing, China; 3 School and Hospital of Stomatology, Peking University, Beijing, China; 4 School of Medicine, Zhejiang University, Hangzhou, China; 5 The Fourth Central Hospital of Baoding City, Baoding, China

**Keywords:** AI-HEALS, breast cancer, Fogg behavior model, mobile health, RCT, self-management

## Abstract

**Background:**

Breast cancer is the most common malignancy among women, and postoperative self-management is critically associated with long-term quality of life. Traditional clinical nursing and mobile health interventions frequently lack behavioral theory support, thereby failing to meet individualized rehabilitation needs. The Fogg Behavior Model (FBM) provides a theoretical framework for precise intervention through the dimensions of motivation, ability, and prompts. Accordingly, this study developed an FBM-based AI-HEALS intervention and will validate its effectiveness via a Randomized Controlled Trial (RCT) to optimize digital transitional care after breast cancer surgery.

**Methods:**

The study will conduct a single-blind, single-center randomized controlled trial in the Fourth Central Hospital of Baoding City, Hebei Province, with female postoperative breast cancer patients aged ≥18 years with stage I-III breast cancer. The control group received routine care, while the intervention group received the AI-HEALS intervention plan in addition to the routine care. Primary outcomes include changes in self-management behavior at baseline and 4, 12, and 24 weeks of follow-up. Secondary outcomes encompass mental health status, self-management efficacy, quality of life, and physiological-related indicators.

**Discussion:**

The aim of this study was to develop an intervention programme for postoperative self-management in breast cancer patients by using the Fogg Behavior Model (FBM) as the theoretical framework and the AI-HEALS system as the intervention platform. Although FBM has shown effectiveness in explaining and promoting health behaviors in various chronic disease populations, systematic studies of its application to postoperative breast cancer self-management are lacking. Our intervention framework aims to stimulate motivation, enhance ability, and provide timely prompts to overcome the limitations of single-component or non-theory-based interventions in complex home-based rehabilitation scenarios. The AI-HEALS system addresses the challenges of traditional mHealth interventions—such as insufficient theoretical grounding, lack of personalisation, and inadequate handling of action triggers—and is expected to improve patients' self-management behaviors, mental health, and quality of life through precise knowledge delivery, dynamic AI-powered question-and-answer support, and full-cycle follow-up management. The development of the AI-HEALS system helps to reduce the burden on healthcare professionals, improve the distribution of healthcare resources, and have potential health economic benefits.

**Clinical trial registration:** https://www.chictr.org.cn/showproj.html?proj=317062, Identifier: ChiCTR2600126533.

## 1 Introduction

Breast cancer is one of the most common malignant tumor among women worldwide and it is a key disease in current cancer prevention and control as well as long-term survival management (1, 2). According to GLOBOCAN's 2022 data, there are about 2,296,800 new cases of breast cancer worldwide. This figure ranks among the top in the total number of cancer cases. With the continuous advancements in screening, surgery, radiotherapy, chemotherapy, endocrine therapy and targeted therapy, the overall survival period of breast cancer patients has continued to increase (3, 4, 67). The focus of breast cancer care has gradually expanded from merely perioperative safety to postoperative rehabilitation, long-term follow-up, and quality management of survival. Chinese breast cancer-related guidelines also indicate that self-management is essential for postoperative rehabilitation of breast cancer (3, 5, 6).

Moreover, the peak age of incidence for Chinese women is relatively earlier, suggesting that long-term postoperative management holds significant public health importance (2, 6–8).

For patients who have undergone breast cancer surgery, discharge from the hospital doesn't mean the end of the rehabilitation process, instead, it marks the beginning of a more complex stage of self-management (9, 10). Patients often need to continuously complete tasks such as wound and drainage observation, upper limb exercise on the affected side, symptom identification and management, medication compliance, appointment scheduling for follow-up visit, weight and sleep management, psychological adjustment, and reconstruction of family roles in their home environment (11–13). Meanwhile, surgical trauma, changes in physical appearance, treatment-related side effects, and uncertainty about recurrence and metastasis can further compound issues such as fatigue, pain, sleep disturbances, anxiety, depression, and fear of disease progression (14). These, in turn, affect patients' quality of life, treatment adherence, and rehabilitation efficiency (15–20). Previous studies have shown that breast cancer survivors may still experience some long-term problems such as fatigue, sleep disorders, anxiety, depression, fear of disease progression and so on. These long-term problems not only affect daily life, but weaken the patients, ability to consistently maintain healthy behaviors (20, 21).

The most crucial challenge in the post-operative care of breast cancer patients does not lie in a single symptom itself, but rather the “long-term rehabilitation behaviors that need to be consistently maintained” are often difficult to consistently occur in real-life situation (22). An existing longitudinal studies in China have shown that the self-management level and quality of life of breast cancer patients after surgery exhibit dynamic changes at different time points (23, 66). The self-management skills do not become firmly established through a single education session; instead, they fluctuate as the postoperative stage progresses, the treatment burden increases, and the family support changes (15, 24). That means that single discharge education sessions of infrequent phone follow-ups are insufficient to meet the frequent, fragmented and dynamically changing needs of patients during the early postoperative rehabilitation period (25).

Digital health tools, such as chatbots and electronic health apps, provide new possibilities for continuous postoperative care. Systematic reviews and meta-analyses suggest that eHealth or remote network interventions have a certain positive impact on outcomes such as quality of life, anxiety, depression, and fatigue in cancer patients (26). Digital health tools also have such advantages such as high accessibility, high scalability, and limited time and space constraints (27–29). Studies targeting the breast cancer population have also shown that intervention based on Wechat or mobile applications can improve quality of life, reduce fear of disease recurrence, enhance self-efficacy, and help patients complete post-treatment rehabilitation tasks (18, 19, 30, 31). However, there are still three disadvantages in the existing evidence. First, many digital interventions lack clear behavior theory support, resulting in an unclear correspondence between content delivery and behavior change mechanisms. This disadvantage makes it difficult to explain why the intervention was effective, for whom it was effective, and through what mechanism it worked (21, 28, 68). Second, some interventions are mianly based on information provision, emphasizing education while relatively weakening the handling of the difficulty in patients' behavior execution, the timing of action triggers, and the management of individual-specific obstacles, thereby affecting the compliance in the real world (32). Third, the current approaches mostly focus on quality of life or a single psychological variable, while paying insufficient attention to “self-management behavior,” which is a core outcome more closely related to the nursing goals (33).

Fogg Behavioral Model (FBM) provides a relatively clear theoretical approach to deal with these problems. According FBM, the occurrence of an action depends on three elements: motivation, ability, and prompt, all of which must be present simultaneously. If the patient knows “what to do” but lacks the motivation, the behavior is difficult to sustain. If the patient “is willing to do” but he is unable to perform the task or finds the task is too complicated, the behavior will be difficult to carry out. If the patient has both the motivation and the ability, but is not promptly reminded, the behavior might be frogotten by him (34). This model is highly suitable for explaining why patients after breast cancer surgery don't consistently carry out rehabilitation behaviors such as wound observation, functional exercise, medication administration, and follow-up visits. It also facilitates the integration of education, question-and-answer, monitoring, push notifications, and human support in the digital platform into a set of actionable behavioral intervention systems.

This integrated role will be undertaken by the AI-HEALS platform (35). This platform does not merely provide health knowledge. Instead, it combines individualized health education and AI-powered question-and-answer support to target three aspects: patient motivation, ability, and prompts. Health education helps patients understand the significance and behavioral benefits of postoperative rehabilitation, thereby enhancing behavioral motivation. AI question-and-answer support helps patients break down complex tasks into actionable steps, thereby enhancing their behavioral capability. Recent studies have also indicated that the future development of digital health interventions for cancer survivors should be more robust, more personalized, and more closely aligned with specific outcomes in intervention design (18, 28, 29, 36).

Based on the above content, this study intends to take FBM as the core theoretical framework, and develop and evaluate an AI-HEALS intervention program specifically for post-operative breast cancer patients (35). We will focus on examining its impact on patients' self-management behaviors, anxiety, depression, self-management efficacy, quality of life, and fear of disease progression (37, 38). This research, on the one hand, can provide a more explanatory and repeatable template for the plan of digital continuation care after breast cancer surgery (19). On the other hand, it also helps to promote the transformation of nursing intervention from “content-oriented” to “behavior mechanism-oriented,” and has strong theoretical significance and practical value.

## 2 Methods

### 2.1 Study design

This study is a single-center, prospective, parallel-controlled, 1:1 allocation, single-blind randomized controlled trail. This study will be carried out at the Fourth Central Hospital of Baoding City, Hebei Province. The design of the plan and the framework of the report should be written in a standardized manner as per requirement of SPIRIT 2025 and CONSORT 2025 (36, 37). This research flowchart is shown in Figure 1.

**Figure 1:**
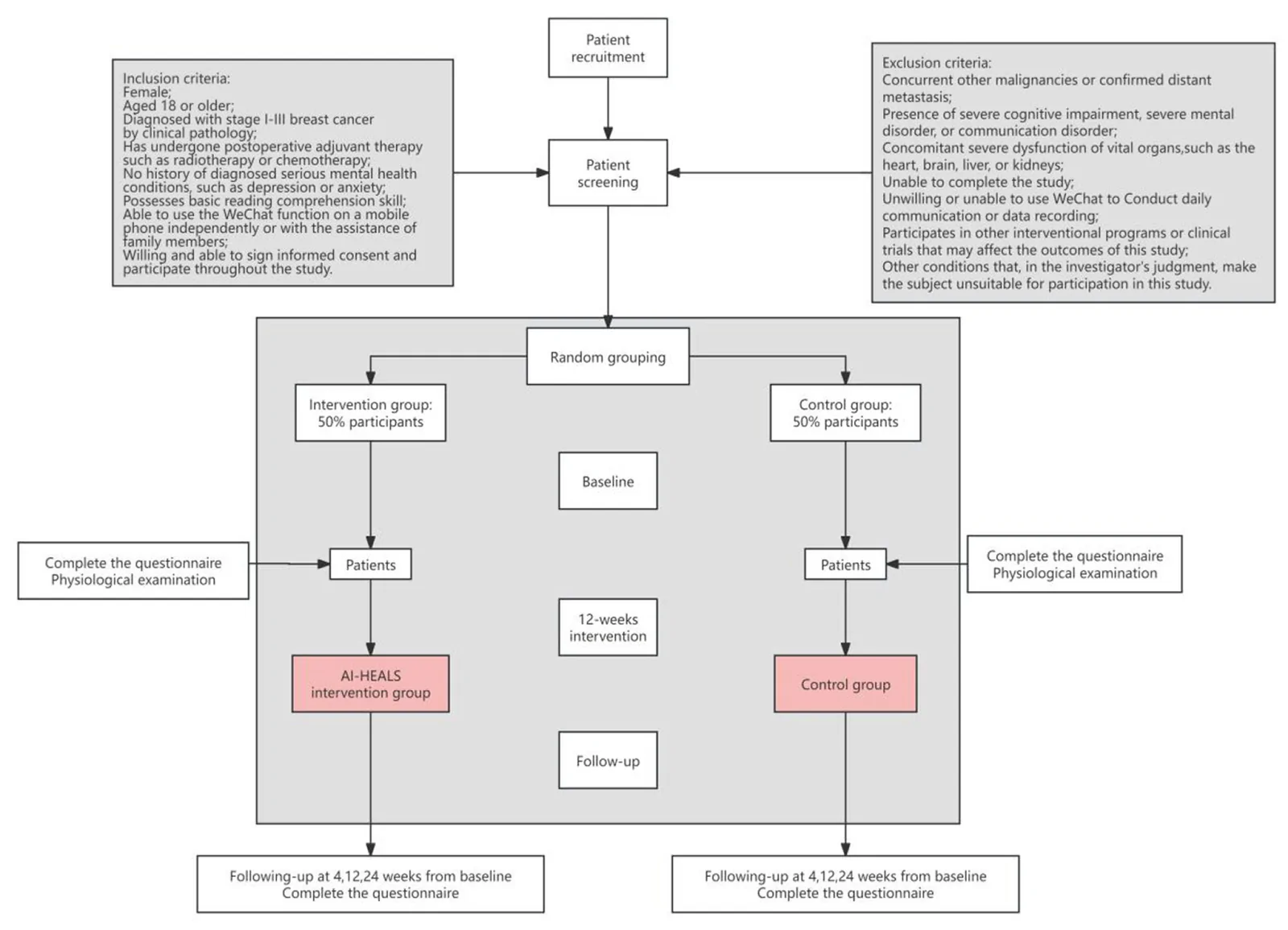
Flow chart of patient recruitment and study implementation.

### 2.2 Randomization and blinding

We used the Excel software for randomization to ensure that the group allocation was fair and without any bias. We have created a new worksheet. The numbers in column A range fromn 1 to N, where N represents the total number of participants. In column B, we use the formula” = RAND ()” to generate random numbers between 0 and 1 for each participant. These random numbers were subsequently corrected by copying and pasting them as “values” to prevent any modifications. Subsequently, the participants were sorted according to random numbers and then divided into groups based on this. The random allocation sequence will be generated by the researchers.

Due to the inherent characteristics of the digital health intervention under investigation, it was not feasible to blind either the study participants or the intervention providers to group allocation. Participants allocated to the intervention group will actively use the AI-HEALS platform, thereby becoming aware of their assignment, while those in the control group will receive usual care without access to the platform. To maintain objectivity and minimize detection bias, the research assistants responsible for outcome data collection and the statistician performing the final data analysis will remain blinded to group assignment. During clinical measurements and questionnaire administration, the research assistants will not be informed of the participants' group allocation. The statistician will analyse the data after de-identification and will not have access to any unblinded allocation information until the entire analysis is fully completed. Only after the final analysis has been documented will the code be broken to reveal group assignments for result interpretation. This approach ensures that the key decision-making points in the trial, outcome assessment and data analysis, remain free of bias.

### 2.3 Study sample

This research intends to continuously recruit eligible breast cancer patients who have undergone surgery at the Breast Surgery Department or Oncology Surgery Department of the Fourth Central Hospital of Baoding City, Hebei Province.

#### 2.3.1 Inclusion criteria

(1) Female; (2) Aged 18 or older; (3) Diagnosed with stage I–III breast cancer by clinical pathology; (4) Has undergone postoperative adjuvant therapy such as radiotherapy or chemotherapy; (5) No history of diagnosed serious mental health conditions, such as depression or anxiety; (6) Possesses basic reading comprehension skill; (7) Able to use the WeChat function on a mobile phone independently or with the assistance of family members; (8) Willing and able to sign informed consent and participate throughout the study.

#### 2.3.2 Exclusion criteria

(1) Concurrent other malignancies or confirmed distant metastasis; (2) Presence of severe cognitive impairment, severe mental disorder, or communication disorder; (3) Concomitant severe dysfunction of vital organs, such as the heart, brain, liver, or kidneys; (4) Unable to complete the study; (5) Unwilling or unable to use WeChat to Conduct daily communication or data recording; (6) Participates in other interventional programs or clinical trials that may affect the outcomes of this study; (7) Other conditions that, in the investigator's judgment, make the subject unsuitable for participation in this study.

### 2.4 Sample size

This study is a RCT. The intervention group is the AI-HEALS intervention group based on the FBM, while the control group is the conventional care group. The “total score of the Cancer Patient Self-Management Assessment Scale” of the study subjects is the main outcome indicator for observation (39). Based on the scale's psychometric properties and previous studies using the CSMAS in breast cancer populations (40, 41), we set the mean difference between the two groups as δ = 10.0, which represents 5.68% of the full scale range (44–220) and is comparable to the established minimal clinically important difference (MCID) thresholds for other breast cancer patient-reported outcome measures [e.g., 7–8 points for FACT-B (42)]. The common standard deviation as σ = 18.0, two-sided test as α = 0.05, power of test as 1–β = 0.80. We use the formula for comparing the means of two independent samples:

After substituting the parameters, *n* = 2 × (1.96 + 0.84)^2^ × 18^2^/10^2^ = 50.8. That means that each group will require at least 51 patients. Taking into account a 20% rate of loss to follow-up, withdrawal or incomplete compliance, we ultimately decided to include 64 cases in each group, resulting in a total sample size of 128 cases.

### 2.5 Recruitment

This research intends to continuously recruit eligible breast cancer patients who have undergone surgery at the Breast Surgery Department or Oncology Surgery Department of the Fourth Central Hospital of Baoding City, Hebei Province. The hospital medical staff will initially screen potential eligible participants during the patients' visits or post-operative follow-ups. The research team will explain in detail to the patients the purpose, methods, potential benefits and risk of the study. Patients who are willing to participate in this study will be officially included in the research after fully understanding the study content and signing the informed consent form.

### 2.6 Informed consent

All participants will sign informed consent forms after being fully informed. The informed consent process will be conducted transparently and openly, strictly adhering to ethical principles. The research team will provide eligible patients with a detailed explanation of the study purpose, procedures, potential risks and benefits, privacy protection measures, as well as specifics regarding data collection, storage, and protection. Patients will be given sufficient time to read the informed consent form, raise questions, and make decisions voluntarily. Patients will be informed that participation in the study is entirely voluntary, that they have the right to withdraw at any stage without giving a reason, and that withdrawal will not affect their subsequent treatment and nursing services. All participants are explicitly informed that the AI tool serves as a supportive aid and does not replace clinical consultation, and are repeatedly encouraged to contact their healthcare providers for any clinical decisions. During the study, platform accounts will be managed using coding, and research data will be accessible only to project members for scientific analysis, with strict protection of personal privacy and data security.

### 2.7 Intervention

#### 2.7.1 Control group

The control group will receive the current routine discharge guidance and postoperative follow-up care provided by their respective departments, which included face-to-face education before discharge, distribution of printed or electronic health education materials, notification of follow-up appointment times, guidance on routine functional exercise, and telephone follow-up when necessary.

#### 2.7.2 Intervention group

In addition to routine care, the intervention group will receive a 12-week, FBM-based AI-HEALS intervention. AI-HEALS utilizes the “Breast Cancer Rehabilitation Manager” platform to promote patients' postoperative rehabilitation behaviors through the three dimensions of motivation, ability, and prompts. Specifically, the platform provides emotional support content, companionship and entertainment functions via the FAQRobot, as well as continuous recording and monitoring of rehabilitation progress and health behaviors. These features help improve patients' attitudes toward rehabilitation, willingness to participate, and self-management confidence, primarily targeting the “motivation” dimension. A health chatbot for postoperative breast cancer rehabilitation, personalized health information push notifications, and professional guidance on wound care, affected limb protection, upper limb functional exercises, lymphedema prevention, nutritional management, and psychological adjustment enable patients to acquire timely knowledge, understand rehabilitation requirements, and master specific operational methods. This primarily targets the “capability” dimension. Automated and personalized information delivery generated based on users' queries and reading behaviors, along with periodic system notifications, mainly acts on the “prompts” dimension. These three factors jointly influence the target behaviors of breast cancer survivors, including self-monitoring of wounds and symptoms, protection of the affected limb, upper limb functional exercise, timely medication adherence, timely follow-up examinations, emotional self-regulation, and active seeking of support. The service consists of four components.

1. Knowledge Base Question and Answer (KBQA) for knowledge about breast cancer surgery rehabilitation. We will encourage participants to use the FAQRobot to obtain accurate and efficient information about post-operative rehabilitation for breast cancer, including post-operative wound care and protection of the affected limb (such as avoiding weight-bearing on the affected limb, measuring blood pressure, drawing blood, etc.,), and upper limb functional exercises (providing recommended exercise methods, intensity and frequency, such as guiding patients to climb walls, grasp balls, stretch, etc.,), and prevention and management of lymphedema (such as identifying early symptoms, skin care, use of pressure sleeves, etc.,), nutrition and dietary management (such as recommended and non-recommended foods during the postoperative recovery period, what nutrients should be supplemented, and how to manage weight, etc.,), and emotional regulation and psychological support (such as how to deal with anxiety, how to build a positive mindset when dealing with depression, etc.,). FAQRobot will eliminate the time and distance limitations of medical consultations and enhance the accessibility of medical services. In addition, this robot also has entertainment functions. It can tell jokes and play games with patients, helping them relax their emotions. Following a review of guidelines, professional literature, and expert consultations, we identified the raw materials and knowledge base for the Breast Cancer Postoperative Rehabilitation and Self-Management KBQA. Subsequently, we integrated this knowledge base into the Doubao Large Language Model developed by ByteDance. Through this approach, the model can learn from the database and provide professional answers to patients directly on the WeChat platform. All responses are derived exclusively from a pre-verified, curated knowledge base. This knowledge base was constructed from clinical guidelines, peer-reviewed literature, and expert consultations. Every piece of information underwent review and approval by specialists in breast surgery and oncology nursing prior to system deployment. The system was implemented using the Doubao platform, a low-code AI development environment that enables knowledge base-grounded retrieval. Rather than relying on the language model's parametric memory, the system retrieves content exclusively from the pre-approved knowledge base before generating each response, thereby minimizing the risk of hallucination or deviation. FAQRobot associates with uses “Breast Cancer Rehabilitation Manager” WeChat platform. The questions raised by the participants and their usage behaviors (such as the time and frequency of usage) will be recorded on the backend, so that we can regularly improve the knowledge graph and evaluate the usage patterns of the participants.
2. Recording and monitoring of physiological indicators and rehabilitation behaviors. Participants will be encouraged to report physiological indicators (e.g., affected limb circumference, weight, blood pressure), upper limb functional recovery (e.g., range of motion, activities of daily living), physical activity (e.g., type, frequency, and duration of rehabilitation exercises), medication usage (e.g., endocrine therapy drugs, analgesics), and emotional status (e.g., self-rated anxiety and depression) by asking questions or providing descriptions within the platform. These self-reported records will be stored electronically and accessible on the back end of the platform, allowing the research team to track long-term rehabilitation progress and changes in self-management behaviors based on the content of participants' inquiries.
3. Medication and rehabilitation support. Participants will be encouraged to note or record the time points they wish to track, such as taking endocrine therapy drugs, checking the circumference of the affected limb, performing rehabilitation training, and attending physical follow-up examinations. This information will be recorded within the platform, facilitating self-scheduled tracking and providing a reference for both participants and the research team when reviewing rehabilitation progress.
4. Manually transmitted personalized information regarding postoperative recovery from breast cancer. Participants will receive manually sent messages weekly regarding breast cancer postoperative rehabilitation and management. These messages cover topics such as postoperative rehabilitation exercises, protection of the affected limb, lymphedema prevention, nutritional support, psychological adjustment, and medication adherence, aiming to enhance health literacy and promote behavioral changes. Additionally, based on the questions posed to the FAQ robot, the research team will manually push further customized articles to participants. Researchers will also periodically log in to the backend to review the number, type, and timing of articles read by participants, allowing them to adjust the content and frequency of information based on individual reading habits.

All user data will be kept confidential. Participants may choose to withdraw from the study at any time. Adverse events related to the intervention (such as pain in the affected limb, skin injury, or exacerbation of lymphedema caused by improper exercise) will be recorded and reported according to local procedures. The operation diagram of the intervention system can be found in Figure 2.

**Figure 2:**
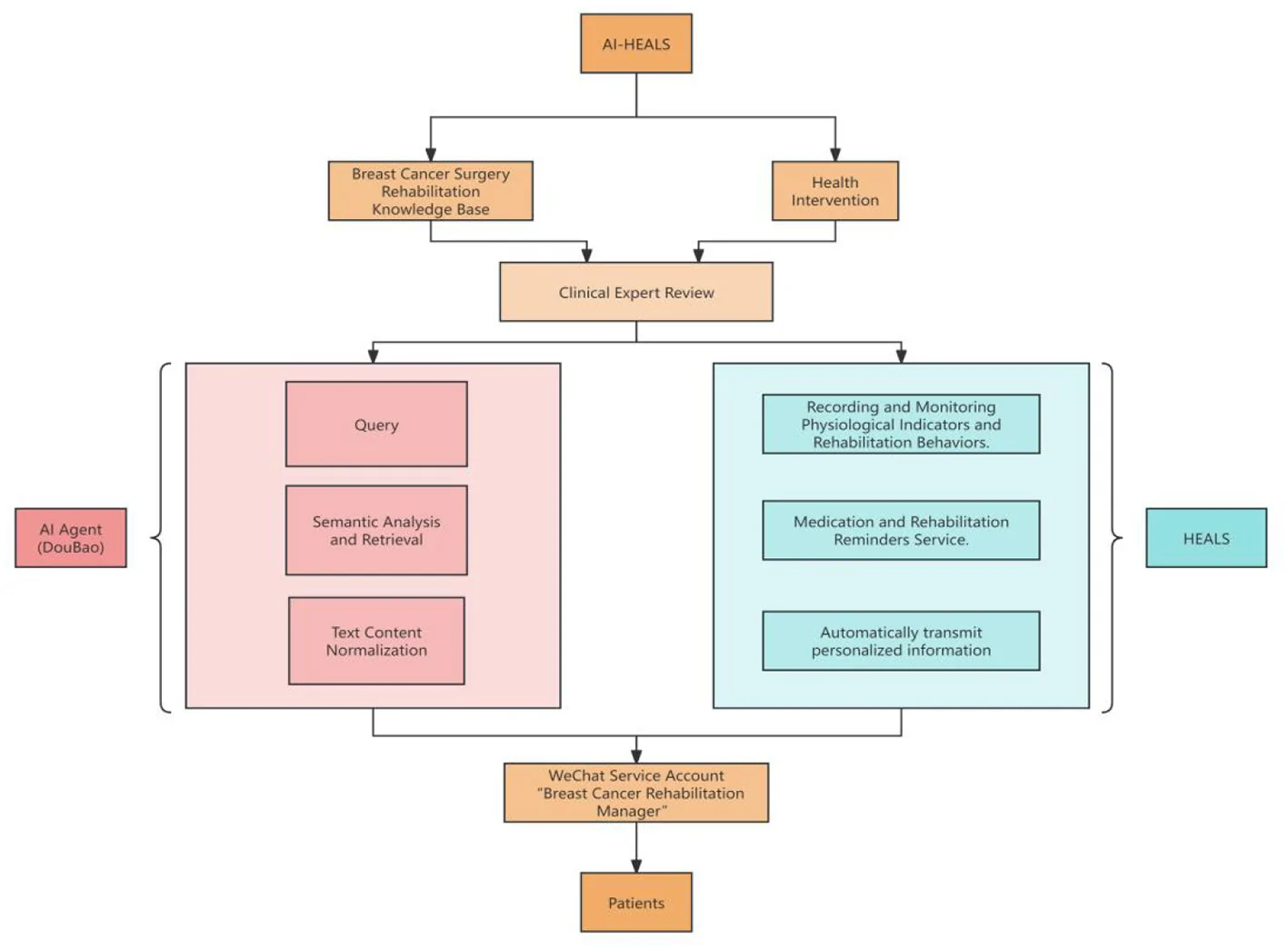
Schematic diagram of the operation of the intervention system.

### 2.8 Quality assurance of study procedures

To ensure participant adherence, this study will develop a standardized intervention manual, standard scripts, and standard operating procedures (SOPs) for platform operations. All research nurses will undergo standardized training and pass an assessment prior to commencing their duties. The health education modules, notification frequency, and alert thresholds will be locked into a specific version prior to the study, and intervention records will be randomly reviewed each month to verify compliance with the protocol.

### 2.9 Outcomes

#### 2.9.1 Primary outcomes

Self-management behaviors: assessed using the Cancer Patient Self-Management Assessment Scale (39). Cancer Patient Self-Management Assessment Scale (CSMAS) is one of the most widely used specialized assessment tools in the field of cancer self-management. The scale comprises six dimensions-daily life management, symptom management, psychological management, communication with healthcare providers, information management, and self-efficacy management-and consists of 44 items. It employs a 5-point Likert scale. Each item is scored on a 5-point scale, with response options of “Never,” “Rarely,” “Sometimes,” “Often,” and “Always,” corresponding to scores of 1, 2, 3, 4, and 5, respectively. A higher score indicates greater self-management ability. The total score ranges from 44 to 220 points, with a Cronbach's alpha of 0.959 (41). Higher scores indicate better self-management.

#### 2.9.2 Secondary outcomes

##### 2.9.2.1 Anxiety and depression

Assessed using the PHQ-4 (43). The abbreviated Patient Health Questionnaire-4 (PHQ-4) is designed to assess patients' symptoms of depression and anxiety over the past 2 weeks. The scale consists of two subscales: the PHQ-2, intended for screening for depression, and the GAD-3 intended for screening for anxiety, comprising a total of four items. Each item is scored on a 4-point scale, where 0 indicates “not at all” and 3 indicates “almost every day.” The total score ranges from 0 to 12, with the depression and anxiety subscales each ranging from 0 to 6, with a Cronbach's alpha of 0.833 (44). A score of 3 or higher suggests a possible anxiety or depressive disorder and requires further evaluation (45).

##### 2.9.2.2 Self-management efficacy

Assessed using the Chinese version of the Cancer Self-Management Efficacy Scale (C-SUPPH), a higher score indicates greater confidence in self-management (46). The Chinese version of the Strategies Used by People to Promote Health (SUPPH) is suitable for assessing self-efficacy levels and evaluating the effectiveness of interventions among patients with various types of cancer (47). The scale originally consisted of 29 items across 4 dimensions but was later revised to 28 items, comprising three dimensions: self-stress management (10 items), self-decision-making (3 items), and positive attitude (15 items). Items are rated on a 5-point scale ranging from 1 (no confidence) to 5 (very confident). The total score ranges from 28 to 140 points, a higher score indicates stronger self-efficacy and greater confidence in disease management among cancer patients. The scale demonstrates excellent reliability and validity, with a Cronbach's α of 0.970 for the total scale and 0.849–0.970 for each dimension. Confirmatory factor analysis has validated its three-factor structure with good fit (48).

##### 2.9.2.3 Quality of life

The Chinese version of the FACT-B will be used to assess quality of life after breast cancer surgery, a higher total score indicates better quality of life (49, 50). The Functional Assessment of Cancer Therapy—Breast (FACT-B) (V4.0) consists of 36 items across 5 domains: Physical Status (7 items), Social/Family Status (7 items), Emotional Status (6 items), Functional Status (7 items), and Additional Concerns (9 items). Scores can be calculated for individual items, each domain, and the total scale. The 36 items of this scale are designed as Likert-type items. When scoring, positive items are directly scored on a scale of 0 to 4, while reverse-scored items (specifically GP1–GP7, GE1, GE3–GE6, B1–B3, and B5–B8) are scored in reverse. The score for each domain is obtained by summing the scores of the items within that domain, and the total scale score is obtained by summing the scores of all domains (51). The Chinese version of the FACT-B scale demonstrates good reliability, validity, responsiveness, and feasibility. The Cronbach's α for the five domains—Physical Status, Social/Family Status, Emotional Status, Functional Status, and Additional Concerns—are 0.84, 0.84, 0.79, 0.83, and 0.61 respectively (50–52).

##### 2.9.2.4 Fear of disease progression

Assessed using the FoP-Q-SF, a higher score indicates a higher level of fear of disease progression (53, 69). The Fear of Progression Questionnaire-Short Form (FoP-Q-SF) consists of 12 items and uses a 5-point Likert scale for self-reporting by patients. The total score ranges from a minimum of 12 to a maximum of 60 points. The Cronbach's alpha for the total scale is 0.883, and the Cronbach's α for the two extracted common factors are 0.829 and 0.812, respectively (54, 55).

##### 2.9.2.5 Fear of cancer recurrence

Assessed using the FCRQ7, a higher score indicates a more severe level of fear (51). The Fear of Cancer Recurrence Questionnaire-7 (FCRQ7) was developed by Professor Gerry Humphris and colleagues at the University of St. Andrews in the United Kingdom to assess cancer patients' fear and anxiety regarding disease recurrence and progression. The scale consists of 7 items; the first 6 items are rated on a 1–5 scale and primarily assess the patient's level of fear. Item 7 is scored on a scale of 1 to 10 and is used to assess the impact of FCR on patients' daily lives and social functioning. The original scale has a Cronbach's α of 0.92; higher scores indicate more severe fear, though no clear cutoff point for severity has been established. This scale demonstrates good reliability and validity, is easy to complete, and is highly practical (56).

##### 2.9.2.6 Comparative prognosis

Rate of distant metastasis, recurrence rate, and overall incidence of complications (collected from hospital medical records during follow-up visits).

### 2.10 Variables measurement

Socio-demographic variables, clinical data, and anthropometric variables will be collected at baseline. Each outcome measure (self-management behaviors, anxiety and depression, self-management efficacy, quality of life, fear of disease progression, and fear of cancer recurrence) will be assessed using the corresponding scales at baseline, 4 weeks after intervention, 12 weeks after intervention (primary endpoint), and 24 weeks after intervention (follow-up) (Table 1).

**Table 1:** The schedule of enrolment, interventions, and assessments.

Study period	Recruitment	Intervention	Follow-up	
Timepoint	0	4 weeks	12 weeks	24 weeks
Eligibility screen	√			
Informed consent	√			
Allocation	√			
Sociodemographic variables	√			
CSMAS	√	√	√	√
PHQ-4	√	√	√	√
C-SUPPH	√	√	√	√
FACT-B	√	√	√	√
FoP-Q-SF	√	√	√	√
FCRQ7	√	√	√	√
DMR, RR, CR			√	√

CSMAS, Cancer Self-Management Assessment Scale; PHQ-4, the abbreviated Patient Health Questionnaire-4; C-SUPPH, the Chinese version of the Strategies Used by People to Promote Health; FACT-B, the Functional Assessment of Cancer Therapy-Breast; FoP-Q-SF, the Fear of Progression Questionnaire-Short Form; FCRQ7, the Fear of Cancer Recurrence Questionnaire-7; DMR, RR, CR, Distance Metastasis Rate, Relapse Rate, Complication Rate.

Socio-demographic variables to be collected include age, educational level, marital status, occupation, monthly per capita household income, and insurance status. Clinical data include tumor size, breast cancer stage, history of neoadjuvant chemotherapy, history of chemotherapy, history of radiotherapy, surgical site, lymph node dissection method, and self-assessment of physical appearance (discrepancy between actual and ideal appearance, and the importance of appearance in daily life). Anthropometric variables include height and weight, and BMI will be calculated.

### 2.11 Statistical analysis

In this study, statistical analyses will be performed using SPSS, R, or SAS. Descriptive statistics will be first conducted for general information and baseline characteristics: continuous variables expressed as mean ± standard deviation or median (interquartile range), and categorical variables presented as frequency and proportion. Comparisons of baseline demographic characteristics between groups will be performed using *t*-tests, the Mann–Whitney *U*-test, the chi-square test, or Fisher's exact test. Changes in scale scores between baseline and endpoint, as well as differences in scale scores between the two groups at baseline and endpoint, will be compared using *t*-tests or non-parametric tests (57, 58).

Missing data will be handled using multiple imputation methods after assessing the mechanisms of missing data; results from the complete-case analysis will be reported concurrently for comparison. All tests will be two-sided, and a *P*-value < 0.05 will be considered statistically significant. Additional analytical methods may be applied based on actual data characteristics, and any such adjustments will be fully reported.

## 3 Study management

### 3.1 Data collection

Prior to the start of the study, all researchers will undergo standardized training, during which experts will train and evaluate team members on operational procedures and technical details to ensure they are familiar with the study protocol, intervention measures, and data collection processes. During the study, the principal investigator will periodically review the research team's work to ensure adherence to the study protocol and proper data collection procedures. Data cleaning and coding will be performed using a double-checking method. A missing data tracking sheet will be established to impute key outcomes where possible, and logic validation rules will be set up to trace back to original records when outliers are detected. The statistical analysis plan will be finalized prior to analysis to avoid result-oriented analysis. The data manager is responsible for overseeing data management to ensure that information is accurate, reliable, and compliant with the study protocol.

### 3.2 Storage and archiving of data

All study data (including the participant identification code list, original questionnaires, intervention records, and laboratory test results) will be archived in the hospital's research archive or a dedicated encrypted database after study completion, in accordance with hospital regulations. Data will be retained for at least 5 years to meet legal and journal publication requirements. Access to the data will be restricted to authorized members of the research team, and any data export will be documented.

### 3.3 Ethics committee

The hospital's Institutional Review Board (IRB) will oversee the progress of this study to ensure it is conducted in accordance with the protocol. The IRB will periodically review the study's implementation. If any deviations from the protocol or issues that may affect participant safety are identified, the IRB may require the study to be suspended or terminated. All proposed study amendments will be submitted to the Ethics Committee for review and approval before implementation.

### 3.4 Confidentiality

When patients submit queries through the WeChat-based “Breast Cancer Rehabilitation Manager” platform, the queries are transmitted to the Doubao API for natural language processing. However, no identifiable patient information (e.g., name, hospital ID, contact details) is included in these API calls—only de-identified query content necessary for generating a response is transmitted. The response generated by the model is then returned directly to the patient through the WeChat platform. All interactions are recorded and subject to daily monitoring and weekly audits by the research team.

Participants' personal information will be strictly protected. During the study, platform accounts will be managed using coding, and research data will be accessible only to project members for scientific analysis. Encryption measures will be applied during data storage and transmission to prevent unauthorized access. No personally identifiable information will be disclosed in any publication of the study results.

### 3.5 Investigator responsibilities

The principal investigator will oversee the entire study process and ensure that all researchers comply with the study protocol and standard operating procedures. Investigators are responsible for ensuring the authenticity and integrity of the data and for reporting study progress to the principal investigator regularly. If issues with data recording or intervention delivery are identified, the research team will promptly correct them.

### 3.6 Protocol amendments

Any amendments to the study protocol must be proposed by the principal investigator and submitted to the Ethics Committee for review and approval. Upon approval, a formal document detailing the changes will be prepared and distributed to all relevant parties. The trial coordinator will document all protocol amendments, which will be signed by the principal investigator; this record will be retained along with other trial documents.

### 3.7 Safety monitoring

The system is equipped with safety alerts that recognize high-risk queries (e.g., those suggestive of infection, severe pain, lymphedema complications, or psychological distress) and directs such queries to the research nurse or attending physician for direct human evaluation, rather than generating AI-based responses.

Although this study involves a low-risk behavioral intervention, safety thresholds will be established for abnormal symptoms and psychological crises. Upon system alerts, a research nurse will conduct a telephone assessment and, if necessary, contact the attending physician or recommend emergency department evaluation. If patients' free-text responses contain explicit expressions of self-harm, suicide, or extreme despair, the department's crisis management protocol will be immediately activated. We have established a psychological crisis response protocol designed to identify patient expressions suggestive of self-harm, suicidal ideation, or extreme emotional distress. Upon identification of such expressions during routine monitoring, the research team will promptly notify the attending physician and initiate a predefined response protocol, comprising a supportive message to the patient and a referral to appropriate mental health resources. Study-related adverse events will be recorded and reported in accordance with hospital regulations.

Additionally, we review AI-patient interactions on a daily basis, with the research team conducting weekly audits of a sample of exchanges. Any identified issues are used to iteratively refine the knowledge base and system prompts.

## 4 Dissemination plans

Upon completion of the study, the research team will provide all participants with a summary of the findings via email to thank them for their support and contributions to the study. The research findings will be submitted to peer-reviewed journals both domestically and internationally and presented at relevant academic conferences to facilitate the exchange and dissemination of the research outcomes. Additionally, the research team will provide the findings and related intervention materials to other healthcare institutions or researchers upon request, with the aim of promoting the application and validation of the AI-HEALS intervention based on the Fogg Behavior Model among a broader population.

## 5 Discussion

Although previous research has demonstrated the potential value of theory-based mobile health interventions in the rehabilitation management of breast cancer patients (18, 19, 27–29), most of these interventions lack a clear behavioral theoretical foundation, resulting in an ambiguous correspondence between intervention content and the mechanisms of behavior change (21, 28). Furthermore, traditional interventions tend to focus primarily on information provision; they pay insufficient attention to the difficulty of behavioral execution, the timing of action triggers, and the handling of individual-specific barriers. On this basis, the present study adopted the Fogg Behavior Model (FBM) as its core theoretical framework to develop and evaluate the AI-HEALS intervention program, aiming to promote postoperative self-management behaviors in breast cancer patients through the synergistic effects of motivation, ability, and prompts. FBM explicitly states that behavior occurs only when motivation, ability, and a prompt are present simultaneously (34). AI-HEALS enhances patients' motivation by improving their understanding of the significance and benefits of postoperative rehabilitation through health education; it improves patients' ability by breaking down complex rehabilitation tasks into actionable steps via AI-powered knowledge base question-and-answer and personalized guidance; and it supports the “prompts” dimension by delivering automated and personalized information based on users' queries and reading behaviors, helping to ensure that actions are taken at appropriate times (35). This design addresses the shortcomings of previous digital interventions, including insufficient theoretical grounding and inadequate handling of individual differences, thereby making the correspondence between intervention measures and behavioral change mechanisms clearer (59).

This study innovatively integrates FBM with the AI-HEALS technology platform. AI-HEALS is not merely a provider of health knowledge; rather, it combines individualized health education and AI-powered question-and-answer support into an actionable behavioral intervention system. Compared with previous studies (18, 19, 30, 31), AI-HEALS not only enhances real-time interactivity but also improves patients' multidimensional self-management abilities through structured educational content and a knowledge-based question-and-answer system, thereby promoting the formation and maintenance of health behaviors. The platform leverages the WeChat service account “Breast Cancer Rehabilitation Manager” to achieve seamless integration between hospital and home settings, aligning with China's “Internet + Healthcare” policy orientation and being particularly suitable for breast cancer patients requiring long-term management. In addition, the platform's backend records patients' questions and usage behaviors, allowing the research team to periodically optimize the knowledge graph and evaluate patients' usage patterns, thus enabling dynamic and precise intervention adjustments.

Despite the relative rigor of our study design, some potential limitations require attention. First, our inclusion criteria require that patients be able to use WeChat functions independently or with family assistance; however, older or less educated patients may face operational difficulties. For this reason, research nurses will provide face-to-face training on system operation at the start of the intervention to ensure that each participant is proficient. Second, this study is being implemented at a single center (the Fourth Central Hospital of Baoding City, Hebei Province), which may limit the representativeness of the sample. Third, a key limitation of this study is its single-blind design, necessitated by ethical and technical constraints. While this design risks performance bias, as staff are aware of group allocations, we will mitigate this through standardized procedures, fixed assessment scripts, and regular audits. Fourth, despite rigorous measures—including physical separation, staff training, and process management—to prevent intergroup “contamination,” we cannot entirely preclude control group patients encountering information about the AI-HEALS system outside the clinical setting. Such contamination would likely dilute the observed treatment effect, meaning our findings should be considered a conservative estimate. Fifth, due to time constraints for participants, we proposed to collect data at baseline and at 4, 12, and 24 weeks of follow-up; however, this follow-up period is still insufficient for assessing long-term efficacy, especially for a chronic process such as post-operative breast cancer rehabilitation that requires continuous management over several years. Sixth, although the development and maintenance of AI-HEALS do not require excessive funding, the updating of the question-and-answer knowledge base and push content still requires continuous follow-up by professionals. This remains a challenge for healthcare workers who are busy with their own work, and system stability (e.g., server response speed) may affect patient experience; however, with the development of AI technology, stability and response speed are expected to gradually improve.

From a health economic perspective, AI-HEALS is expected to provide important support for post-operative breast cancer rehabilitation management in China. By reducing repetitive workload through semi-automated education, it can alleviate the pressure on healthcare human resources. According to China's healthcare resource distribution, high-quality medical resources are mainly concentrated in tertiary hospitals, while patients in primary-level hospitals and remote areas lack access to follow-up resources. AI-HEALS, built on the WeChat platform, has the advantages of low cost, wide coverage, and high accessibility, making it particularly suitable for resource-limited areas or patients with restricted mobility. It is anticipated that this intervention will improve patients' self-management behaviors, mental health, and quality of life, thereby reducing additional medical expenditures caused by complications or recurrence (60, 61). Breast cancer is the most common malignancy among women in China, and the economic burden of long-term management cannot be ignored (3, 7). Therefore, the health economic benefits of this study warrant further in-depth analysis.

The goal of this study was to construct an innovative self-management intervention program for post-operative breast cancer patients by integrating the Fogg Behavior Model with AI-HEALS technology (62). The rationality and validity of the theoretical framework, the precision of the technology platform, and the adaptability of the implementation strategy of this program will set a new benchmark for behavioral intervention research in chronic diseases (63, 64). If the results of the trial are positive, it is expected that this intervention model can be extended to other cancer types requiring long-term self-management (e.g., post-operative colorectal cancer patients or breast cancer patients receiving long-term endocrine therapy) or other chronic diseases (e.g., coronary heart disease, diabetes), promoting the application of the “motivation-ability-prompt” triple-driven paradigm of health management in a wider range of disease management fields (23, 65).

## References

[1] Benitez Fuentes JD Morgan E de Luna Aguilar A Mafra A Shah R Giusti F . Global stage distribution of breast cancer at diagnosis: a systematic review and meta-analysis. JAMA Oncol. (2024) 10:71–8. doi: 10.1001/jamaoncol.2023.4837 PMC10636649 37943547

[2] Dessie ZG Zewotir T. Global determinants of breast cancer mortality: a comprehensive meta-analysis of clinical, demographic, and lifestyle risk factors. BMC Public Health. (2025) 25:2640. doi: 10.1186/s12889-025-24036-w PMC12323182 40760691

[3] Bray F Laversanne M Sung H Ferlay J Siegel RL Soerjomataram I . Global cancer statistics 2022: GLOBOCAN estimates of incidence and mortality worldwide for 36 cancers in 185 countries. CA Cancer J Clin. (2024) 74:229–63. doi: 10.3322/caac.21834 38572751

[4] Dickinson K Sharma A Agnihotram RV Altuntur S Park M Meterissian S . Circulating tumor DNA and survival in metastatic breast cancer: a systematic review and meta-analysis. JAMA Netw Open. (2024) 7:e2431722. doi: 10.1001/jamanetworkopen.2024.31722 PMC11378006 39235812

[5] Perdomo M Davies C Levenhagen K Ryans K Gilchrist L. Patient education for breast cancer-related lymphedema: a systematic review. J Cancer Surviv. (2023) 17:384–98. doi: 10.1007/s11764-022-01262-4 PMC9546750 36207626

[6] Jia J Shan ZH Zhao YH Geng Y. Chinese expert consensus on community rehabilitation of upper limb dysfunction in patients after breast cancer surgery (2026 edition). Chin J Med. (2026) 61:413–9. doi: 10.3969/j.issn.1008-1070.2026.04.005

[7] The Society of Breast Cancer China Anti-Cancer Association. breast oncology group of the oncology branch of the Chinese medical association. guidelines for breast cancer diagnosis and treatment by china anti-cancer association (2024 edition). China Oncol. (2023) 33:1092–187. doi: 10.19401/j.cnki.1007-3639.2023.12.004

[8] Beebeejaun Y Athithan A Copeland TP Kamath MS Sarris I Sunkara SK. Risk of breast cancer in women treated with ovarian stimulation drugs for infertility: a systematic review and meta-analysis. Fertil Steril. (2021) 116:198–207. doi: 10.1016/j.fertnstert.2021.01.044 34148584

[9] Tan S Yang J Guo G Hong S Guo L Situ H. The risk of dementia in breast cancer survivors: a meta-analysis of observational studies. Ann Med. (2025) 57:2529579. doi: 10.1080/07853890.2025.2529579 PMC12269053 40671353

[10] Bu X Jiang L Leung DYP. Fear of cancer recurrence prevalence and its associated factors among family caregivers of women with breast cancer: a systematic review and meta-analysis. J Clin Nurs. (2025) 34:2510–24. doi: 10.1111/jocn.17680 PMC12181156 39994915

[11] Gieni M Avram R Dickson L Farrokhyar F Lovrics P Faidi S . Local breast cancer recurrence after mastectomy and immediate breast reconstruction for invasive cancer: a meta-analysis. Breast. (2012) 21:230–6. doi: 10.1016/j.breast.2011.12.013 22225710

[12] Chen X Wang Q Zhang Y Xie Q Tan X. Physical activity and risk of breast cancer: a meta-analysis of 38 cohort studies in 45 study reports. Value Health. (2019) 22:104–28. doi: 10.1016/j.jval.2018.06.020 30661625

[13] Laustsen-Kiel CM Hansen L Ørholt M Zhang SM Krezdorn N Vester-Glowinski PV . The impact of breast reconstruction compared with no reconstruction on breast cancer-related lymphedema: a systematic review and meta-analysis. Surgery. (2025) 187:109649. doi: 10.1016/j.surg.2025.109649 40953534

[14] Sebri V Martino ML Singer J Pravettoni G. (2026) A tailored psychological intervention based on self-defining autobiographical memories and body image in breast cancer survivors: a commentary. HPR. 14: e81240042. doi: 10.14440/hpr.0189

[15] Zhang X Du X Wang R Yang X Guo R Su L . A longitudinal study of self-management and quality of life in patients after breast cancer surgery. J Nurs Sci. (2021) 36:41–3. doi: 10.3870/j.issn.1001-4152.2021.06.041

[16] Mosconi P Folli S Miceli R Scrudato S Gennaro M Tinè G . Health-related quality of life and fear of recurrence after early breast cancer treatment: results from a cross-sectional survey. Breast. (2026) 85:104647. doi: 10.1016/j.breast.2025.104647 PMC12670931 41270536

[17] Wang LP Yao LH Wang D Lim GH Almaghrabi MY Shen H . A WeChat-based nursing intervention program improves the postoperative rehabilitation of breast cancer patients: results from a randomized controlled trial. Gland Surg. (2024) 13:383–94. doi: 10.21037/gs-24-40 PMC11002481 38601277

[18] Zhou K Wang W Zhao W Li L Zhang M Guo P . Benefits of a WeChat-based multimodal nursing program on early rehabilitation in postoperative women with breast cancer: a clinical randomized controlled trial. Int J Nurs Stud. (2020) 106:103565. doi: 10.1016/j.ijnurstu.2020.103565 32272281

[19] Pizot C Boniol M Mullie P Koechlin A Boniol M Boyle P . Physical activity, hormone replacement therapy and breast cancer risk: a meta-analysis of prospective studies. Eur J Cancer. (2016) 52:138–54. doi: 10.1016/j.ejca.2015.10.063 26687833

[20] Jiménez-Díaz A Escrivá-Martínez T Herrero R Baños R. Evidence-based digital health interventions for breast cancer survivors'care: an umbrella review. Internet Interv. (2025) 41:100846. doi: 10.1016/j.invent.2025.100846 PMC12242455 40641665

[21] Tian L Wen Y Li T Liu C Fan J. Effects of using app-based interventions on quality of life among breast cancer patients: a systematic review with meta-analysis. Ann Med. (2025) 57:2499027. doi: 10.1080/07853890.2025.2499027 PMC12057781 40327523

[22] Xu A Wang Y Wu X. Effectiveness of e-health based self-management to improve cancer-related fatigue, self-efficacy and quality of life in cancer patients: systematic review and meta-analysis. J Adv Nurs. (2019) 75:3434–47. doi: 10.1111/jan.14197 31566769

[23] Li R Liang N Bu F Hesketh T. The effectiveness of self-management of hypertension in adults using mobile health: systematic review and meta-analysis. JMIR Mhealth Uhealth. (2020) 8:e17776. doi: 10.2196/17776 PMC7148553 32217503

[24] Jathanna N Strachan K Erhayiem B Kamaruddin H Swoboda P Auer D . The nottingham ischaemic cardiovascular magnetic resonance resource (NotIs CMR): a prospective paired clinical and imaging scar database-protocol. J Cardiovasc Magn Reson. (2023) 25:69. doi: 10.1186/s12968-023-00978-1 PMC10680206 38008732

[25] Wen J Hui SS Chin EC Yang Y Sit CH. The effectiveness of mind-body exercise on health-related quality of life and mental health during and after breast cancer treatment: an umbrella review of meta-analyses for randomized controlled trials. Worldviews Evid Based Nurs. (2025) 22:e70008. doi: 10.1111/wvn.70008 PMC11911294 40090889

[26] Wen T Chen C Ren W Hu S Zhao X Zhao F . Effect of electronic health (eHealth) on quality of life in women with breast cancer: a systematic review and meta-analysis of randomized controlled trials. Cancer Med. (2023) 12:14252–63. doi: 10.1002/cam4.6094 PMC10358200 37199350

[27] Tian L Wen Y Li J Guan J Li T Fan J. Effects of remote web-based interventions on the physiological and psychological states of patients with cancer: systematic review with meta-analysis. JMIR Mhealth Uhealth. (2025) 13:e71196. doi: 10.2196/71196 PMC12203031 40505121

[28] Kim SM Kim DS Jang Y Kim MK Yu ES Han DH . Evaluating the effectiveness of a mobile app for breast cancer self-management on self-efficacy: nonrandomized intervention trial. JMIR mHealth and uHealth. (2025) 13:e63989. doi: 10.2196/63989 PMC11982755 40138696

[29] Cheng ASK Liu X Ng PHF Kwok CTT Zeng Y Feuerstein M. Breast cancer application protocol: a randomised controlled trial to evaluate a self-management app for breast cancer survivors. BMJ Open. (2020) 10:e034655. doi: 10.1136/bmjopen-2019-034655 PMC7337895 32624468

[30] Kirsch EP Kunte SA Wu KA Kaplan S Hwang ES Plichta JK . Digital health platforms for breast cancer care: a scoping review. J Clin Med. (2024) 13:1937. doi: 10.3390/jcm13071937 PMC11012307 38610702

[31] Shi N Ching Wong AK Yuet Wong FK Zhang N Zhu W Shen K . Feasibility of a mobile health app-based self-management program for Chinese patients with breast cancer receiving chemotherapy: a randomized controlled pilot study. Digit Health. (2024) 10:20552076241231560. doi: 10.1177/20552076241231560 PMC10896051 38410790

[32] Galiano-Castillo N Ariza-García A Cantarero-Villanueva I Fernández-Lao C Díaz-Rodríguez L Legerén-Alvarez M . Telehealth system (e-CUIDATE) to improve quality of life in breast cancer survivors: rationale and study protocol for a randomized clinical trial. Trials. (2013) 14:187. doi: 10.1186/1745-6215-14-187 PMC3704734 23799886

[33] Reme SE Munk A Holter MTS Falk RS Jacobsen HB. Pre- and post-operative psychological interventions to prevent pain and fatigue after breast cancer surgery (PREVENT): Protocol for a randomized controlled trial. PLoS ONE. (2022) 17:e0268606. doi: 10.1371/journal.pone.0268606 PMC9269362 35802618

[34] Fogg BJ. A behavior model for persuasive design. In: Proceedings of the 4th international conference on persuasive technology. New York, NY: ACM (2009). p. 1–7. doi: 10.1145/1541948.1541999

[35] Wu Y Min H Li M Shi Y Ma A Han Y . Effect of Artificial Intelligence-based Health Education Accurately Linking System (AI-HEALS) for Type 2 diabetes self-management: protocol for a mixed-methods study. BMC Public Health. (2023) 23:1325. doi: 10.1186/s12889-023-16066-z PMC10334542 37434126

[36] Chan AW Boutron I Hopewell S Moher D Schulz KF Collins GS . SPIRIT 2025 statement: updated guideline for protocols of randomised trials. Lancet. (2025) 405:e19–27. doi: 10.1016/S0140-6736(25)00770-6 42290521

[37] Hopewell S Chan AW Collins GS Hróbjartsson A Moher D Schulz KF . CONSORT 2025 statement: updated guideline for reporting randomised trials. BMJ. (2025) 388:e081123. doi: 10.1136/bmj-2024-081123 PMC11995449 40228833

[38] Liu L Chen M. Evaluation of the application effect of psychological behavior remote intervention guided by health belief model and artificial intelligence in patients with gastric cancer. Nurs Pract Res (2022) 19:1181-4. (in Chinese)

[39] Cheng L L Sun Y Q. Development and validation of cancer patient self-management assessment scale. Chin J Nurs. (2017) 52:1082–7. doi: 10.3761/j.issn.0254-1769.2017.09.014

[40] Li Y Zhang X Wang HL Du X. Investigation on the status quo of self-management in breast cancer patients after radical mastectomy. Tianjin J Nurs. (2021) 29:636–41. doi: 10.3969/j.issn.1006-9143.2021.06.002

[41] Tang L Quan XF Yan XL Wang YN. Correlation between hardiness and self-management level in postoperative patients with renal cell carcinoma. J Nurs Adm. (2018) 18:638. doi: 10.3969/j.issn.1671-315x.2018.09.008

[42] Eton DT Cella D Yost KJ Yount SE Peterman AH Neuberg DS . A combination of distribution- and anchor-based approaches determined minimally important differences (MIDs) for four endpoints in a breast cancer scale. J Clin Epidemiol. (2004) 57:898–910. doi: 10.1016/j.jclinepi.2004.01.012 15504633

[43] Kroenke K Spitzer RL Williams JBW Löwe B. An ultra-brief screening scale for anxiety and depression: the PHQ-4. Psychosomatics. (2009) 50:613–21. doi: 10.1016/S0033-3182(09)70864-3 19996233

[44] Weihs KL Wiley JF Crespi CM Krull JL Stanton AL. Predicting future major depression and persistent depressive symptoms: development of a prognostic screener and PHQ-4 cutoffs in breast cancer patients. Psycho-Oncol. (2017) 27:420–6. doi: 10.1002/pon.4472 PMC6531034 28600855

[45] Thuillard S Augsburger N Batziou S Bruni M Fatio L Martinez N . Validation of a French version of the DERS-SF and creation of a super short form (DERS-F SSF) for measuring emotion regulation difficulties. Sci Rep. 15:40702 (2025). doi: 10.1038/s41598-025-24281-4 PMC12630587 41258295

[46] Qian HJ Yuan CR. Reliability and validity of Chinese version of strategies used by people to promote health. Chin J Nurs. (2011) 46:87–9. (in Chinese)

[47] Lev EL Owen SV. A measure of self-care self-efficacy. Res Nurs Health. (1996) 19:421–9. 10.1002/(SICI)1098-240X(199610)19:5<421::AID-NUR6>3.0.CO;2-S 8848626

[48] Yuan C Qian H Wang J Lev EL Yuan A Hinds PS. Factorial structure of a scale: strategies used by people to promote health–chinese version. Cancer Nurs. (2015) 38:E13–20. doi: 10.1097/NCC.0000000000000151 24945261

[49] Wan CH Zhang DM Tang XL Zhang WL Li WH Ren HX . Revision of the Chinese version of the FACT-B for patients with breast cancer. Chin Ment Health J. (2003) 17:298–300. (in Chinese)

[50] Wan CH Zhang DM Tang XL Zhang WL Li WH Ren HX . Introduction on measurement scale of quality of life for patients with breast cancer: Chinese version of FACT-B. China Cancer. (2002) 11:318–20. doi: 1004-0242(2002)06-0318-03. (in Chinese)

[51] Martino ML Capasso M Lemmo D Donizzetti AR Freda MF Caso D. The moderating role of emotion regulation on the relationship between cancer fatalism and intentions to participate in female cancer screening. HPR. (2026) 14:e81240049. doi: 10.14440/hpr.0371

[52] Sansalone E Zucca A Duncan MJ Morris O Smith SR Robinson S . Study protocol of a pilot randomised controlled trial assessing the feasibility and acceptability of RecoverEsupport: a digital health intervention to enhance recovery in women undergoing surgery for breast cancer. BMJ Open. (2025) 15:e093869. doi: 10.1136/bmjopen-2024-093869 PMC11808865 39920075

[53] Ortlipp LC Buckeridge EM Milne SC. Effectiveness of telehealth versus face-to-face appointments in identifying complications following breast cancer surgery. Supp Care Cancer. (2025) 33:1062. doi: 10.1007/s00520-025-10113-8 41239022

[54] Wu QY Ye ZX Li L Liu PY. Reliability and validity of Chinese version of fear of progression questionnaire-short form for cancer patients. Chin J Nurs. (2015) 50:1515–9. (in Chinese)

[55] Silva Ferreira I Carvalho Fernandes AF Moura Barbosa Castro RC Rodrigues Bezerra A Velasco Yanez RJ. Telenursing in the sexual function of women with breast cancer: a study protocol. Medicine. (2022) 101:e31449. doi: 10.1097/MD.0000000000031449 PMC9704939 36451379

[56] Zhang YF Tan XM Sun HW Sun ZH Zhang JY Liu T . Chinese version of fear of cancer recurrence questionnaire for cancer patients: a preliminary study[J]. Chin J Hyg Rescue. (2019) 5:157–63. (in Chinese)

[57] Kruse CS Pacheco GJ Vargas B Lozano N Castro S Gattu M. Leveraging telehealth for the management of breast cancer: a systematic review. Healthcare. (2022) 10:2015. doi: 10.3390/healthcare10102015 PMC9602569 36292461

[58] Mozer CADN Gonçalves JDC Dos Santos LS Furieri LB Fioresi M. Telenursing practice in the care of surgical cancer patients: a scoping review protocol. BMJ Open. (2023) 13:e074502. doi: 10.1136/bmjopen-2023-074502 PMC10668273 37989371

[59] Zhou Y Li SJ Huang RQ Ma HM Wang AQ Tang XY . Behavior change techniques used in self-management interventions based on mhealth apps for adults with hypertension: systematic review and meta-analysis of randomized controlled trials. J Med Internet Res. (2024) 26:e54978. doi: 10.2196/54978 PMC11538878 39437388

[60] Wu Y Zhang J Ge P Duan T Zhou J Wu Y . Application of chatbots to help patients self-manage diabetes: systematic review and meta-analysis. J Med Internet Res. (2024) 26:e60380. doi: 10.2196/60380 PMC11653048 39626235

[61] Schulman-Green D Jaser SS Park C Whittemore R. A metasynthesis of factors affecting self-management of chronic illness. J Adv Nurs. (2016) 72:1469–89. doi: 10.1111/jan.12902 PMC4891247 26781649

[62] Yoon M Lee JH Kim IC Lee JH Kim MN Kim HL . Smartphone app for improving self-awareness of adherence to edoxaban treatment in patients with atrial fibrillation (adhere-app trial): randomized controlled trial. J Med Internet Res. (2024) 26:e65010. doi: 10.2196/65010 PMC11621717 39570659

[63] Melo Sierra N Hernández Rincón EH Osorio Betancourt GA Ramos Chaparro PA Diaz Quijano DM Barbosa SD . Use of artificial intelligence in the management of stroke: scoping review. Front Radiol. (2025) 5:1593397. doi: 10.3389/fradi.2025.1593397 PMC12141347 40487839

[64] Singleton AC Raeside R Hyun KK Partridge SR Di Tanna GL Hafiz N . Electronic health interventions for patients with breast cancer: systematic review and meta-analyses. J Clin Oncol. (2022) 40:2257–70. doi: 10.1200/JCO.21.01171 PMC9273371 35500200

[65] Zaghloul H Fanous K Ahmed L Arabi M Varghese S Omar S . Digital health literacy in patients with common chronic diseases: systematic review and meta-analysis. J Med Internet Res. (2025) 27:e56231. doi: 10.2196/56231 PMC12377790 40854231

[66] Lu R Yang Z Miao J Xu Q Zhang L. Latent profile analysis of self-management and its association with quality of life differences in patients with cancer treated with immune checkpoint inhibitors. Asia Pac J Oncol Nurs. (2025) 12:100687. doi: 10.1016/j.apjon.2025.100687 PMC12017969 40271525

[67] Rajan KK Fairhurst K Birkbeck B Novintan S Wilson R Savović J . Overall survival after mastectomy versus breast-conserving surgery with adjuvant radiotherapy for early-stage breast cancer: meta-analysis. BJS Open. (2024) 8:zrae040. doi: 10.1093/bjsopen/zrae040 PMC11100524 38758563

[68] Guo XH Wang KL Wu YY Hui H. Application of perioperative nursing guided by knowledge behavior model in patients undergoing colorectal cancer surgery. Int Med Health Guid News. (2024) 30:2787–91. doi: 10.3760/cma.j.issn.1007-1245.2024.16.031

[69] Mahendran R Liu J Kuparasundram S Griva K. Validation of the english and simplified mandarin versions of the fear of progression questionnaire-short form in Chinese cancer survivors. BMC Psychol. (2020) 8:10. doi: 10.1186/s40359-020-0374-0 PMC6995061 32005291

